# Author Correction: Multilayer networks reveal the spatial structure of seed-dispersal interactions across the Great Rift landscapes

**DOI:** 10.1038/s41467-018-06669-1

**Published:** 2018-10-02

**Authors:** Sérgio Timóteo, Marta Correia, Susana Rodríguez-Echeverría, Helena Freitas, Ruben Heleno

**Affiliations:** CFE—Centre for Functional Ecology, Department of Life Sciences, University of Coimbra, Calçada Martim de Freitas, 3000-456 Coimbra, Portugal

Correction to: *Nature Communication* ; 10.1038/s41467-017-02658-y; published online 10 January 2018

The original version of this article contained Figshare links in the Code availability statement that were not functional. The correct Figshare links to MATLAB scripts and R code used in this study are 10.6084/m9.figshare.4955651 and 10.6084/m9.figshare.4836383, respectively. These errors have now been corrected in both the PDF and HTML versions of the article.

