# Supplementary material for: Multilayer networks reveal the spatial structure of seed-dispersal interactions across the Great Rift landscapes
Source: Nat Commun. 2018 Jan 10;9:140. doi: 10.1038/s41467-017-02658-y (PMC5762785; doi:10.1038/s41467-017-02658-y)
Supplement: Supplementary file 1 — Supplementary Information [file 41467_2017_2658_MOESM1_ESM.pdf]

Supplementary Information

Supplementary Methods

DNA barcoding of seeds

DNA was extracted from seeds using the DNeasy Plant Mini Kit (Qiagen) following the manufacturer instructions. The DNA samples were subjected to an additional step of purification with Phenol: Chloroform: Isoamyl alcohol. DNA was re-suspended in 40 µl of elution buffer and kept at 20 ºC. Two chloropastid loci (the psbA-trnH intergenic spacer, and the trnL intron and trnL-F intergenic spacer) were amplified using a Hot Start Taq Master Mix (QIAGEN) as described in[1]. Amplification was performed in 25 µl containing 1 µl of DNA and 1 µl of each primer. Conditions of the PCR were as follows: 95ºC (15min); 94ºC (1min); then 30 cycles for trnL-F and 35 cycles for psbA at 94ºC (1min)/ 50ºC (1min)/ 72ºC (1min), and a final extension at 72°C (10 min). The PCR products were purified using ExoSAP-IT (Affymetrix), and sequenced in a Sanger ABI 3730xl at GATC Biotech (Germany). The sequences were compared with the available online databases using BLAST[2]. The species were identified based on the best BLAST matches and the list of plant species known for the Gorongosa National Park.

Multilayer modularity

Modularity is a structural pattern of interactions between nodes of a network whereby a group of species – a module or community, interact more frequently than expected among them than with other groups of species[6,7]. A multilayer approach to modularity allows the identification communities that span across multiple layers of the network, which can be important to the structural unity of the whole network[8]. We used a modularity quality function that uses a “generalized Louvain” method to community finding[9,10]. The Louvain method for the identification of communities progresses in two iterative phases: in the first phase, all nodes are considered one-by-one and assigned to a specific set of nodes – community, until a configuration is reached that maximizes the modularity quality function. In the second phase, the communities previously found are now used as nodes of a reduced network, and the same procedure is repeated until no further increase in modularity is detected[11]. This is a popular locally-greedy method for modularity-optimization as it is fast and delivers reliable results[12,13]. Following[14], we changed the contribution of the original standard null-model for unipartite networks[7]: $\gamma_s \frac{k_{is}k_{js}}{2m_s}$, i.e. the expected interaction frequency of any two nodes *i* and *j* within layer *s*, on original code of this function (available at

http://netwiki.amath.unc.edu/GenLouvain/GenLouvain), to: $\gamma_s \frac{k_{is} d_{js}}{m_s}$, reflecting the bipartite nature of the network:

$$Q_{multilayer} = \frac{1}{2\mu} \sum_{ijsr} \left[\left(A_{ijs} - \gamma_s \frac{k_{is} d_{js}}{m_s} \right) \delta_{sr} + \delta_{ij} C_{jsr} \right] + \delta(g_{is}, g_{jr}),$$

where A_{ijs} is the weight of the intra-layer edge between nodes *i* and *j* within layer *s*; C_{jsr} is a tensor element giving the weight of the inter-layer between node *j* and its replica on layers *r* and *s* (given the categorical nature of the multilayer coupling in spatial multilayer networks all values $C_{jsr} > 0$, and it is assumed to be equal for any inter-layer coupling, $C_{jsr} = \omega$); γ_s is the resolution parameter for layer *s*; k_{is} and d_{js} are the degrees of plant *i* and dispersers *j* within layer *s*, respectively; m_s is the total edge weight of layer *s*; g_{is} and g_{jr} are the set of nodes forming the communities that contain the nodes-layer (*i,s*) and (*j,s*), respectively; the Kronecker delta between indices *x* and *y* is denoted as δ_{xy} (this will be 1 for $x = y$ and 0 for $x \neq y$), and $2\mu = \sum_{ijs} A_{ijs}$[9].
The "generalized Louvain" methods requires the specification of two parameters: the resolution limit γ; and the inter-layer coupling ω. The resolution limit γ defines the detail to which the network will be resolved into communities, and can be seen as the importance given to the null model relative to the empirical network[12]. We used the default resolution parameter value of $\gamma = 1$[9,12]. The choice of the coupling parameter ω is a matter of intense investigation, and takes a value of either 0 or ω[12]. When $\omega = 0$ it is equivalent to optimizing the modularity for each layer independently, where any node never belongs to the same community across the different layers, i.e. communities are not persistent across the multilayer network. If however $\omega > 0$, and as it increases, nodes are less likely to belong to different communities, which tend to span across the different layers of the network, and can assume different values of each pair of layers depending on the importance of the coupling between those pairs of layers[9,12].

Versatility

To assess the importance of nodes to the structure we calculated centrality for each node accounting for the multilayer nature of our network, defined by the animal-plant interaction in each of the habitats of Gorongosa. This allows to identify the most important nodes – versatile species, in our system[15]. We used a widely used measure of centrality based on Google's PageRank[16], which is a random walk centrality measure corresponding to the path taken by a walker moving between adjacent nodes, with the importance of each node being calculated recursively by the sum of the

importance of all nodes connected to it. PageRank centrality was extended to the case of multilayer networks by allowing “teleportation” of nodes between any layers of the network[15].

Multistrength

Node multistrength measures the strength of a node as the combined weight of its connections, across the different layers of a network[17,18], and expresses the importance of a node to the community of nodes with which it interacts in the multilayer network. Two concepts are important to understand multistrength, namely: multidegree and multilink. Multidegree is the number of links in which a node participates, and it is an extension of node degree for monolayers[17,18]. A multilink is defined as the set of links that connect two nodes in different layers of a network[17,18]: $\vec{m}$ = m_1,m_2,…m_α, …m_M), with each m_α accepting either of two values $m_\alpha = 1$ or 0, defining the set of links between any two nodes in different layers, and in any layer α if $m_\alpha = 1$. It is now introduced the multi-adjacency matrices $A^{\vec{m}}$ where elements $A_{ij}^{\vec{m}} = 1$ if a multilink $\vec{m}$ exists between nodes *i* and *j*, or zero if no link exists:

$$A_{ij}^{\vec{m}} = \prod_{ij}^{\vec{m}} \left[a_{ij}^{\alpha} m_\alpha + \left(1 + a_{ij}^{\alpha}\right) + (1 - m_\alpha)\right],$$

where a_{ij}^{α} is the weight of the link between nodes *i* and *j* in layer α. Any *i* and *j* pair of nodes must satisfy the condition:

$$\sum_{\vec{m}} A_{ij}^{\vec{m}} = 1.$$

Multidegree $\vec{m}$ of any node *i*, $k_i^{\vec{m}}$ is definded as the total number of multilinks $\vec{m}$ incident on node *i*:

$$k_i^{\vec{m}} = \sum_{j=1}^{N} A_{ij}^{\vec{m}}.$$

Then, species multistrength $s_{i,\alpha}^{\vec{m}}$ measures the total weights of the links incident on a node in a given layer which forms a multilink of type $\vec{m}$[17,18]:

$$s_{i,\alpha}^{\vec{m}} = \sum_{j=1}^{N} a_{ij}^{\alpha} A_{ij}^{\vec{m}}.$$

Supplementary Tables

Supplementary Table 1 – Sampling completeness of animal species and plant species. The estimated number of species (S_{est}) was calculated using the non-parametric estimator Chao2[19], and is compared with the observed number of species (S_{obs}). In brackets is present the proportion (%) of S_{obs} in relation to S_{est}.

	Grassland		Transition forest		Mixed forest		Miombo	
	S_{est}	S_{obs}	S_{est}	S_{obs}	S_{est}	S_{obs}	S_{est}	S_{obs}
Animal species	19.5	14 (72%)	27.3	16 (59%)	32.5	21 (65%)	15.9	12 (76%)
Plant species	49.5	29 (59%)	81.0	42 (52%)	219.1	69 (32%)	82.2	24 (29%)

Supplementary Table 2 – Differences in animal richness, plant richness, and number of interactions among the main habitats of Gorongosa. When the overall G-test for detected a significant difference, the results of pair-wise G-tests are shown.

Variable/G-test	Pairwise G-test: p value			
Animal richness: G = 1.836, df = 3, p = 0.607				
Plant richness: G = 9.395, df = 3, p = 0.025		Grassland	Transition forest	Mixed forest
	Transition forest	0.258		
	Mixed forest	0.003	0.071	
	Miombo	0.541	0.097	0.001
No. of Interactions: G = 139.64, df = 3, $p < 2.2e^{-16}$				
	Transition forest	$1.4e^{-09}$		
	Mixed forest	$< 2.0e^{-16}$	$4.5e\text{-}^{05}$	
	Miombo	0.290	$4.3e\text{-}^{07}$	$< 2.0e^{-16}$

Supplementary Table 3 – Results of the generalized linear mixed model (Gamma family) fitted to dispersers specialization (d') by habitat type, with animal species as a random factor. Model fit assessed with the Akike's Information Criterion (AIC) against a reduced model, which only included the intercept.

	Parameter	Estimate ± SEM	t- test	*P*
Dispersers specialization (d')	Intercept	0.743 ± 0.073	10.204	$< 2^{e-16}$
	Habitat (Transition forest)	- 0.052 ± 0.057	- 0.910	0.363
	Habitat (Mixed forest)	- 0.062 ± 0.057	- 1.078	0.281
	Habitat (Miombo)	0.018 ± 0.065	0.274	0.784
	Habitat (overall effect)	X^2 = 2.487, df = 3, p = 0.478		
	X^2 = 2.338, 3 df, p = 0.505<br>$AIC_{reduced}$ = - 16.02; AIC_{model}= -12.36			

Supplementary Table 4 – Versatility, specialization (d'), multistrength, and number of habitats where each disperser species is present.

Species	Multilayer Versatility	Aggregated Versatility	d'	Multistrength	N. Habitats
Papio ursinus	1.000	1.000	0.320	52	4
Loxodonta africana	0.750	0.665	0.483	38	4
Cercopithecus pygerythrus	0.608	0.465	0.426	30	4
Civettictis civetta	0.545	0.384	0.622	26	4
Phacochoerus africanus	0.496	0.268	0.340	21	2
Aepyceros melampus	0.467	0.216	0.711	19	3
Hystrix africaeaustralis	0.466	0.255	0.777	21	3
Redunca arundinum	0.446	0.195	0.669	18	3
Chlorocichla flaviventris	0.438	0.059	0.835	13	1
Andropadus importunus	0.438	0.059	0.390	13	1
Ourebia ourebi	0.432	0.054	0.889	13	2
Herpestidae (Mongoose)	0.432	0.054	0.065	13	1
Hippotragus niger	0.429	0.052	0.828	13	1
Oriolus larvatus	0.427	0.052	0.619	13	1
Kobus ellipsiprymnus	0.426	0.178	0.578	18	3
Numida meleagris	0.426	0.078	0.472	14	1
Connochaetes taurinus	0.425	0.050	0.058	13	1
Cephalophus natalensis	0.425	0.052	0.691	13	2
Tragelaphus sylvaticus	0.423	0.053	0.979	13	3
Corythaixoides concolor	0.417	0.050	0.507	13	1
Otolemur crassicaudatus	0.417	0.050	0.084	13	1
Genetta tigrina	0.406	0.074	0.658	14	2
Potamochoerus larvatus	0.404	0.100	0.493	15	2
Pycnonotus tricolor	0.401	0.126	0.832	16	3
Tragelaphus strepsiceros	0.398	0.073	0.856	14	2
Tragelaphus angasii	0.396	0.119	0.776	16	3

Supplementary Figures

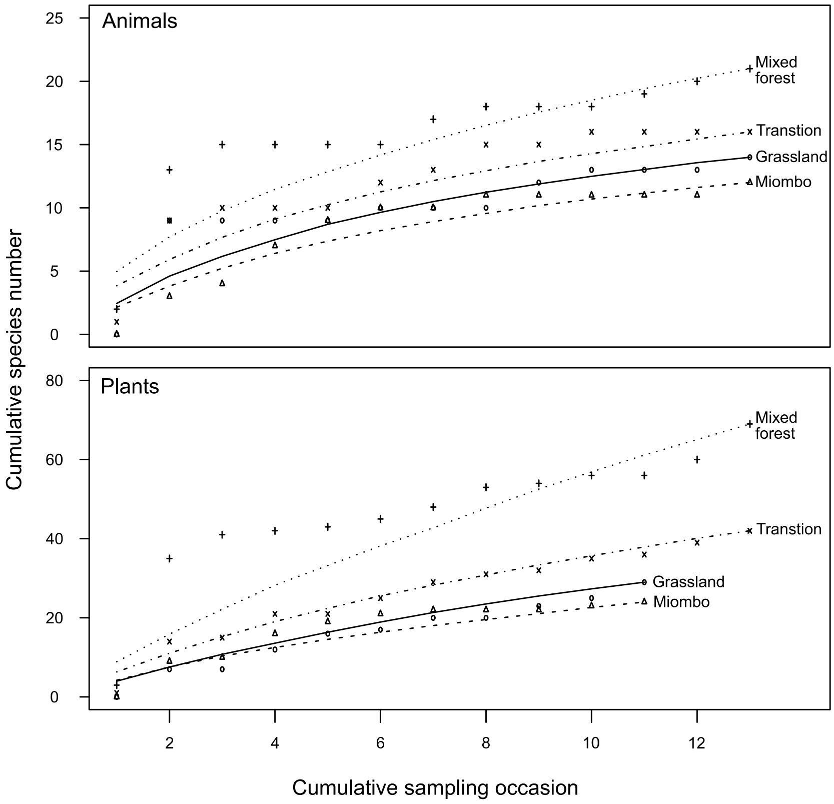

Supplementary Figure 1 - Accumulation curves for animal species (top) and plant species (bottom) in the seed-dispersal network of each habitat of Gorongosa (symbols represent the actual data points for each of the 13 sampling occasions, except for Grassland and Miombo where no interactions were detected in two sampling occassions).

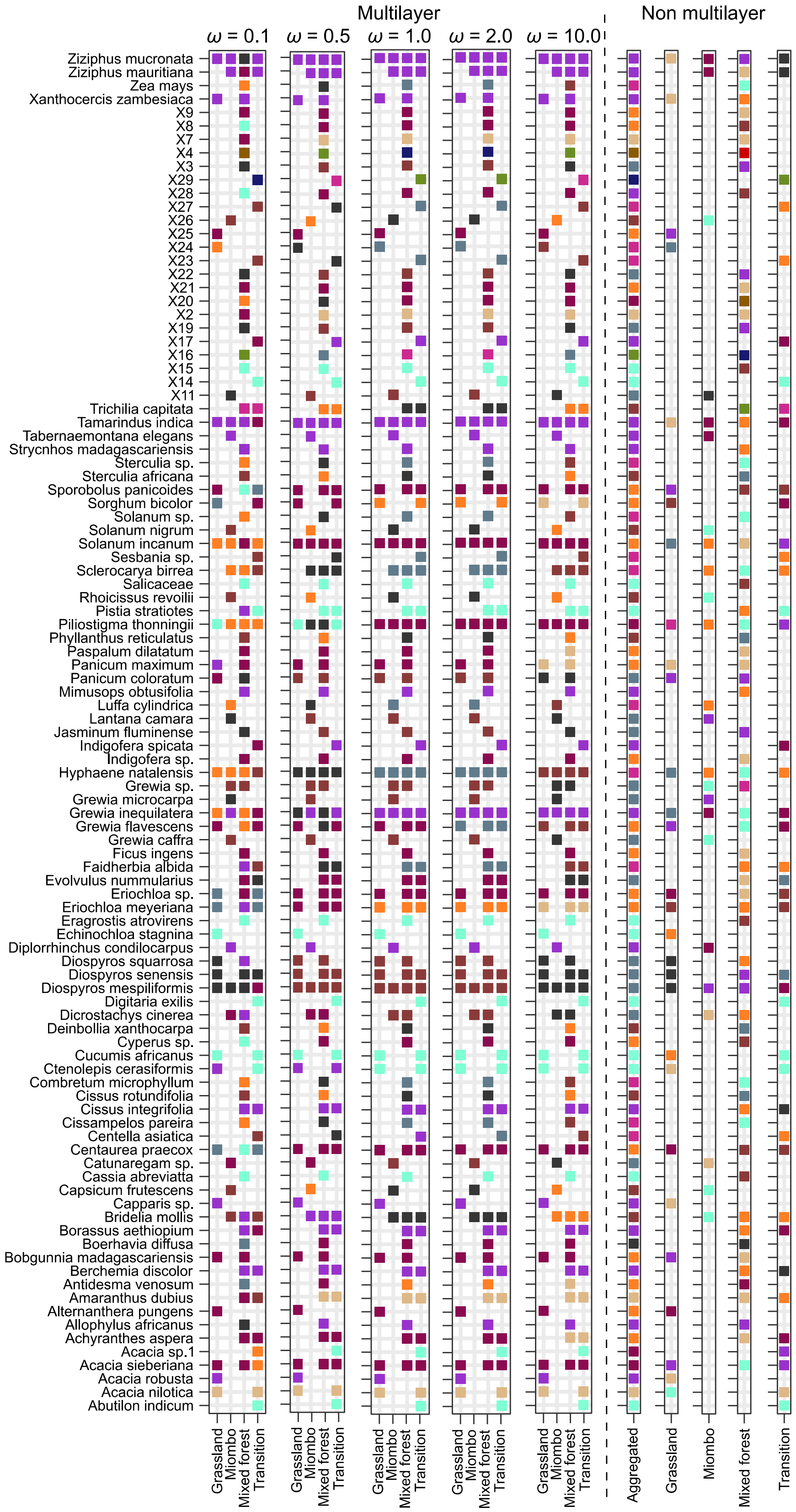

Supplementary Figure 2 - Module affiliation of plant species in the spatial multilayer network of Gorongosa, for five different inter-layer edge strengths (left block), and for the monolayer networks: aggregated and individual habitats (right block). For each case, it was used the run with the highest maximized modularity. Within each network different colours represent different modules. Colours in different blocks are independent.

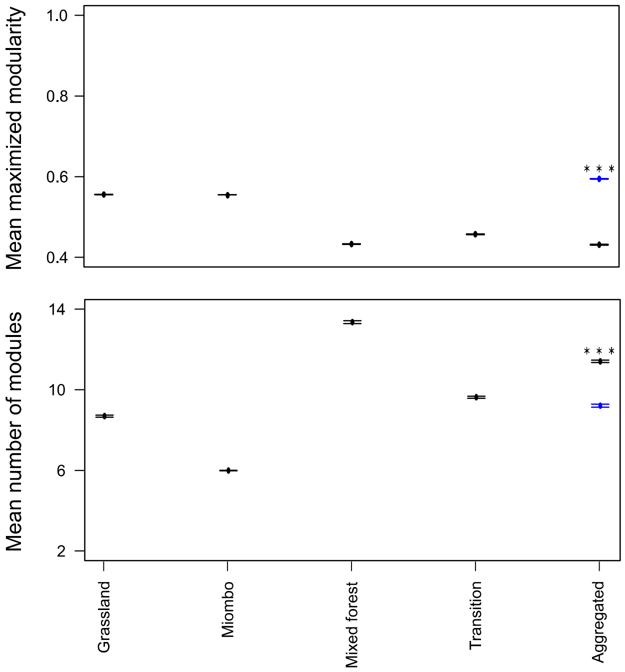

Supplementary Figure 3 - Modularity and number of modules of the observed network in each habitat of Gorongosa, and in the aggregated network (black symbols). The aggregated network pools together all animal-plant interactions regardless of their habitat. Modularity and number of modules predicted by the intra-layer null model are shown for the aggregated network (blue symbols). Values presented as the mean (± SEM) of 100 runs. The significance of the observed modularity was compared against the distribution of the modularity of the null networks. Significance of the number of modules of the observed network was assessed against the null networks with a one-sample t-test.
*** p <0.001.

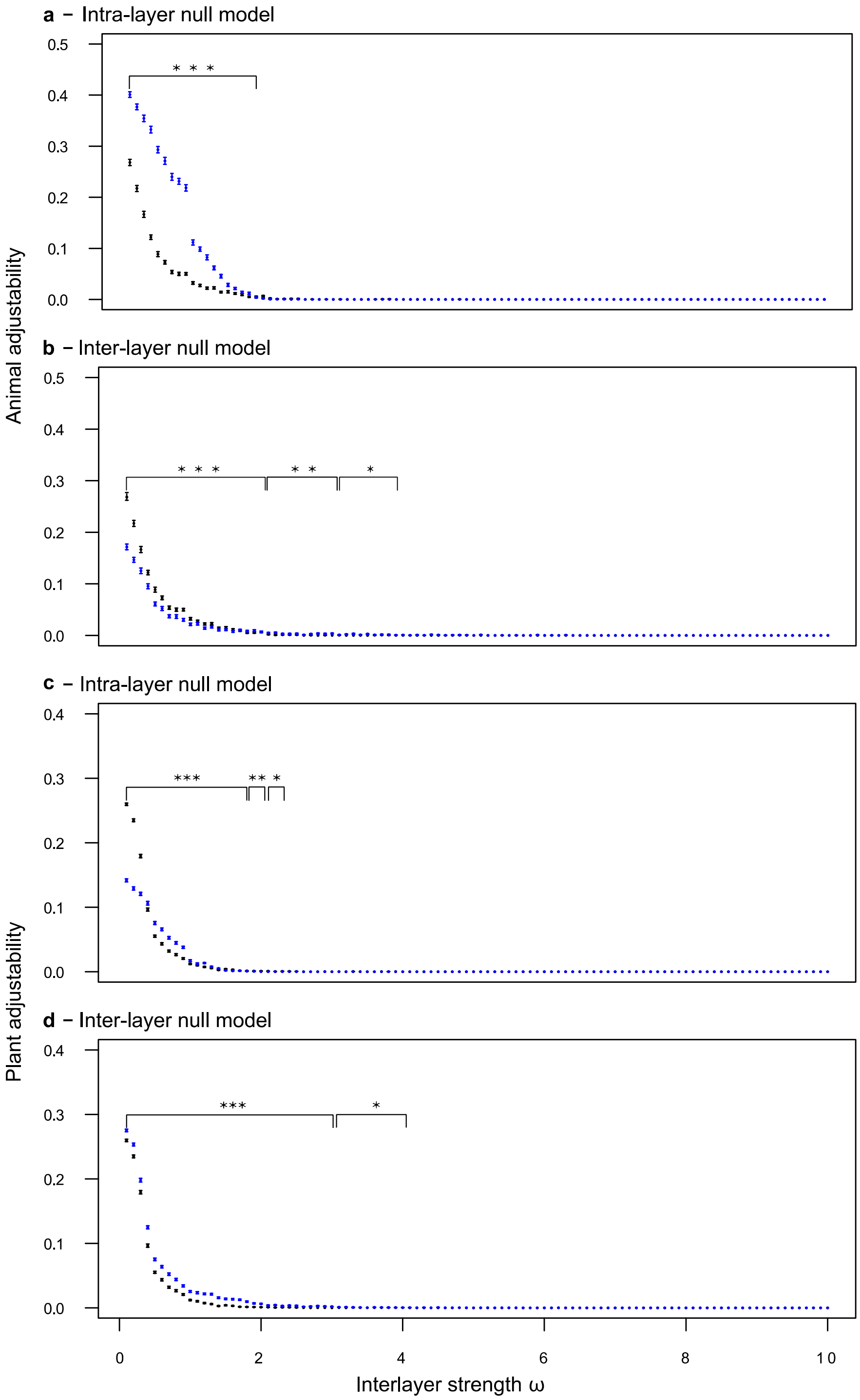

Supplementary Figure 4 - Adjustability (proportion of species that change module affiliation across habitats at least once) of animal (a and b) and plant species (c and d). The observed adjustability (black symbols) is compared against two null models (blue symbols)): intra-layer null model (a and c) and inter-layer null model (b and d). Values presented as the mean (± SEM) of 100 runs of the modularity function, for each interlayer strength. Significance of adjustability in the observed network was assessed against the null networks with an one-sample t-test. * $p < 0.050$, ** $p < 0.010$, *** $p < 0.001$. Beyond the statisitically significant results, t-tests were not statistically significant, or could not be performed due to constancy of data . Full results presented in Supplementary Data 1.

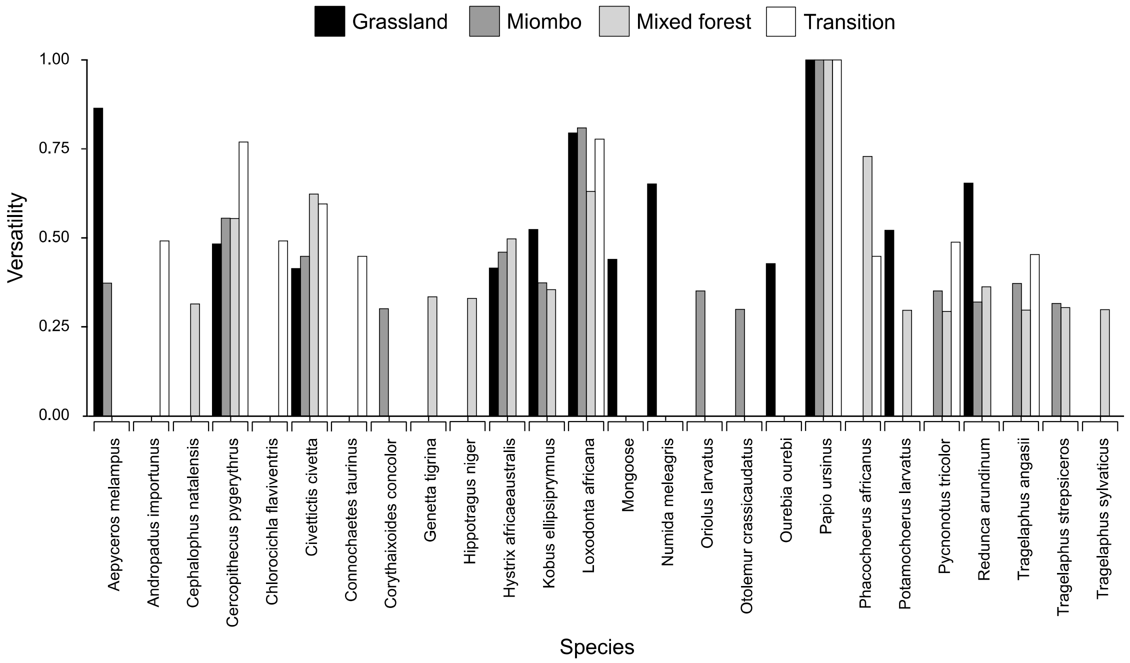

Supplementary Figure 5 - Animal species versatility in the network of each individual habitat.

Supplementary References

1. Kress, W. J. & Erickson, D. L. A two-locus global DNA barcode for land plants: The coding rbcL gene complements the non-coding trnH-psbA spacer region. *PLoS One* **2,** e508 (2007).
2. Altschul, S. F. *et al.* Gapped BLAST and PSI-BLAST: A new generation of protein database search programs. *Nucleic Acids Res.* **25,** 3389–3402 (1997).
3. Chao, A. On the estimation of the number of classes in a population. *Scand. J. Stat.* **11,** 265–270 (1984).
4. Colwell, R. K. & Coddington, J. A. Estimating terrestrial biodiversity through extrapolation. *Philos. Trans. R. Soc. B Biol. Sci.* **345,** 101–118 (1994).
5. Colwell, R. K. EstimateS: Statistical estimation of species richness and shared species from samples. Version 9. (2013).
6. Barber, M. J. Modularity and community detection in bipartite networks. *Phys. Rev. E - Stat. Nonlinear, Soft Matter Phys.* **76,** 1–9 (2007).
7. Girvan, M. & Newman, M. E. J. Community structure in social and biological networks. *Proc. Natl. Acad. Sci. U. S. A.* **99,** 7821–7826 (2002).
8. Pilosof, S., Morand, S., Krasnov, B. R. & Nunn, C. L. Potential parasite transmission in multi-host networks based on parasite sharing. *PLoS One* **10,** 1–19 (2015).
9. Mucha, P. J., Richardson, T., Macon, K., Porter, M. A. & Onnela, J.-P. Community structure in time-dependent, multiscale, and multiplex networks. *Science (80-.).* 876–878 (2010). doi:10.1126/science.1184819
10. Jutlla, I. S., Jeub, L. G. & Mucha, P. J. A generalized Louvain method for community detection implemented in MATLAB. (2014).
11. Blondel, V. D., Guillaume, J.-L., Lambiotte, R. & Lefebvre, E. Fast unfolding of communities in large networks. *J. Stat. Mech. Theory Exp.* **10008,** 6 (2008).
12. Bazzi, M. *et al.* Community detection in temporal multilayer networks, and its application to correlation networks. *Multiscale Model. Simul.* **14,** 1–41 (2016).
13. Lancichinetti, A. & Fortunato, S. Community detection algorithms: A comparative analysis. *Phys. Rev. E - Stat. Nonlinear, Soft Matter Phys.* **80,** 1–11 (2009).
14. Pilosof, S., Porter, M. A., Pascual, M. & Kéfi, S. The multilayer nature of ecological networks. *Nat. Ecol. Evol.* **1,** 101 (2017).
15. De Domenico, M., Sole-Ribalta, A., Omodei, E., Gomez, S. & Arenas, A. Ranking in interconnected multilayer networks reveals versatile nodes. *Nat. Commun.* **6,** 6868 (2015).
16. Brin, S. & Page, L. The anatomy of a large-scale hypertextual web search engine. *Comput. Networks* **56,** 3825–3833 (2012).

17. Menichetti, G., Remondini, D., Panzarasa, P., Mondragón, R. J. & Bianconi, G. Weighted multiplex networks. *PLoS One* **9,** 6–13 (2014).
18. Boccaletti, S. *et al.* The structure and dynamics of multilayer networks. *Phys. Rep.* **544,** 1–122 (2014).
19. Chao, A. Estimating the Population Size for Capture-Recapture Data with Unequal Catchability. *Biometrics* **43,** 783 (1987).
