# Supplementary material for: Multilayer networks reveal the spatial structure of seed-dispersal interactions across the Great Rift landscapes
Source: Nat Commun. 2018 Jan 10;9:140. doi: 10.1038/s41467-017-02658-y (PMC5762785; doi:10.1038/s41467-017-02658-y)
Supplement: Supplementary file 3 — Description of Additional Supplementary Files [file 41467_2017_2658_MOESM3_ESM.pdf]

Description of Additional Supplementary Files

File Name: Supplementary Data 1

Description: Full results of the comparison of the observed multilayer network descriptors against those predicted by null models.
